# Epitope mapping of severe acute respiratory syndrome coronavirus 2 neutralizing receptor binding domain-specific monoclonal antibodies

**DOI:** 10.3389/fmed.2022.973036

**Published:** 2022-09-06

**Authors:** Faezeh Maghsood, Mohammad Mehdi Amiri, Amir-Hassan Zarnani, Vahid Salimi, Gholam Ali Kardar, Jalal Khoshnoodi, Maryam Mobini, Hengameh Ahmadi Zare, Abbas Ghaderi, Mahmood Jeddi-Tehrani, Sylvie Schmidt, Géraldine Laumond, Christiane Moog, Fazel Shokri

**Affiliations:** ^1^Department of Immunology, School of Public Health, Tehran University of Medical Sciences, Tehran, Iran; ^2^Department of Virology, School of Public Health, Tehran University of Medical Sciences, Tehran, Iran; ^3^Immunology, Asthma and Allergy Research Institute, Children’s Medical Center, Tehran University of Medical Sciences, Tehran, Iran; ^4^Shiraz Institute for Cancer Research, School of Medicine, Shiraz University of Medical Science, Shiraz, Iran; ^5^Monoclonal Antibody Research Center, Avicenna Research Institute, Academic Center for Education, Culture and Research (ACECR), Tehran, Iran; ^6^Laboratoire d’ImmunoRhumatologie Moléculaire, Institut National de la Santé et de la Recherche Médicale (INSERM) UMR_S 1109, Institut Thématique Interdisciplinaire (ITI) de Médecine de Précision de Strasbourg, Transplantex NG, Faculté de Médecine, Fédération Hospitalo-Universitaire OMICARE, Fédération de Médecine Translationnelle de Strasbourg (FMTS), Strasbourg, France

**Keywords:** COVID-19, monoclonal antibodies, neutralization, omicron, SARS-CoV-2

## Abstract

Severe acute respiratory syndrome coronavirus 2 (SARS-CoV-2) is the causative agent of the outbreak led to the coronavirus disease 2019 (COVID-19) pandemic. Receptor binding domain (RBD) of spike (S) protein of SARS-CoV-2 is considered as a major target for immunotherapy and vaccine design. Here, we generated and characterized a panel of anti-RBD monoclonal antibodies (MAbs) isolated from eukaryotic recombinant RBD-immunized mice by hybridoma technology. Epitope mapping was performed using a panel of 20-mer overlapping peptides spanning the entire sequence of the RBD protein from wild-type (WT) Wuhan strain by enzyme-linked immunosorbent assay (ELISA). Several hybridomas showed reactivity toward restricted RBD peptide pools by Pepscan analysis, with more focus on peptides encompassing aa 76–110 and 136–155. However, our MAbs with potent neutralizing activity which block SARS-CoV-2 spike pseudovirus as well as the WT virus entry into angiotensin-converting enzyme-2 (ACE2) expressing HEK293T cells showed no reactivity against these peptides. These findings, largely supported by the Western blotting results suggest that the neutralizing MAbs recognize mainly conformational epitopes. Moreover, our neutralizing MAbs recognized the variants of concern (VOC) currently in circulation, including alpha, beta, gamma, and delta by ELISA, and neutralized alpha and omicron variants at different levels by conventional virus neutralization test (CVNT). While the neutralization of MAbs to the alpha variant showed no substantial difference as compared with the WT virus, their neutralizing activity was lower on omicron variant, suggesting the refractory effect of mutations in emerging variants against this group of neutralizing MAbs. Also, the binding reactivity of our MAbs to delta variant showed a modest decline by ELISA, implying that our MAbs are insensitive to the substitutions in the RBD of delta variant. Our data provide important information for understanding the immunogenicity of RBD, and the potential application of the novel neutralizing MAbs for passive immunotherapy of SARS-CoV-2 infection.

## Introduction

Severe acute respiratory syndrome coronavirus 2 (SARS-CoV-2) was first identified in Wuhan, China in December 2019 (1). As of the 24th of April 2022, there were over 500 million global cases of coronavirus disease 2019 (COVID-19) and more than 6 million deaths worldwide (2). SARS-CoV-2, known as the third highly pathogenic human CoV belonging to the lineage B beta-coronaviruses, is a zoonotic enveloped virus containing a positive-sense single-stranded RNA, presumably originated from bats due to sharing 96% genome sequence identity with RaTG13, a bat-derived SARS-like CoV (3, 4).

The genome of SARS-CoV-2 encodes several structural and non-structural proteins. Homotrimeric spike (S) glycoprotein on the viral surface is involved in cell attachment, membrane fusion, and viral entry (5, 6). The S protein with a length of 1,273 amino acids (aa), is a clove-shaped, type I transmembrane protein consisting of a signal peptide (1–13), S1 subunit (14–685), and S2 subunit (686–1,273). The S1 subunit is composed of the N-terminal domain (NTD) (18–305), the C-terminal receptor binding domain (RBD) (329–528), subdomain-1 (SD1) (529–589), and SD2 (590–686) (7). The RBD consists of two sub-domains, including a core sub-domain composed of a β-sheet with five antiparallel strands (β1–β4, and β7) in the inner side of the spike protein and receptor-binding motif (RBM) extending from the core sub-domain and consisting of β5 and β6 strands (8, 9). The RBM is responsible for virus binding to its receptor, the angiotensin-converting enzyme-2 (ACE2), by forming a surface to cradle the N-terminal α-helix of ACE2 expressed on the host cell surface (7). The S2 subunit consists of the upstream helix (UH) (687–819), N-terminal fusion peptide (FP) (820–846), heptapeptide repeat sequence 1 (HR1) (912–985), SD3 (1,072–1,139), stem helix (SH) (1,139–1,163), HR2 (1,163–1,212), TM domain (1,213–1,237), and intracellular domain (1,238–1,273). The S2 subunit plays an important role in mediating fusion of viral membrane with host cell membrane and virus entry into target cells (7, 10). The S protein of SARS-CoV-2 shares about 76% amino acid sequence homology with that of SARS-CoV and both use ACE2 as a receptor for viral entry in a similar way (11, 12). Also, the core sub-domain of RBD, rather than RBM, is more conserved between SARS-CoV-2 and SARS-CoV viruses (identity of 86.3% for the core sub-domain versus 46.7% for the RBM sub-domain) (13). Notwithstanding the critical function of both S1 and S2 subunits in viral entry, it has been shown that anti-S1 antibodies bind to the S protein and neutralize the virus more efficiently than anti-S2 antibodies, presumably due to lack of a major neutralizing region on the S2 subunit (14). Despite less conserved residues and high mutation rates in the RBD rather than in other parts of the S protein, RBD is still considered the most important region of SARS-CoV-2 to be targeted by neutralizing antibodies due to difficult access to and tight folding of fusion domains (15).

Neutralizing monoclonal antibodies (MAbs) have been considered as one of the most promising approaches to target SARS-CoV-2 surface protein as a prophylactic/therapeutic alternative of COVID-19 convalescent plasma for the passive immunotherapy (16–19), since they have been effective against other viruses such as rabies, HIV, RSV, EBOV, SARS-CoV, and MERS-CoV (20, 21). Accordingly, considerable efforts have been made to produce MAbs capable of neutralizing SARS-CoV-2. Until now, U.S. Food and Drug Administration (FDA) or European Medicines Agency (EMA) have not approved neutralizing MAbs for COVID-19 infection, although a number of MAbs have been authorized for emergency use as prophylaxis in adults and children at high risk of severe COVID-19, including sotrovimab, REGN-CoV-2, bebtelovimab, and the combination of bamlanivimab and etesevimab (22–26). Considering the emergence of newly fast-spreading variants and reduced or lack of efficacy of a number of neutralizing MAbs, there is an urgent need for development of efficient neutralizing MAbs that cross-neutralize various variants to control SARS-CoV-2 infection and/or disease progression. In this study, we produced a panel of MAbs directed against the RBD of SARS-CoV-2 using hybridoma technology. These MAbs were then extensively characterized and their neutralization potency against different variants of SARS-CoV-2 was investigated *in vitro*.

## Materials and methods

### Sources of cell lines, antigens, antibodies, and reagents

Sp2/0-Ag14 (murine myeloma cell line) was purchased from the National Cell Bank of Iran (NCBI) (Pasteur Institute, Tehran, Iran). ACE2-expressing HEK293T cell line used for pseudovirus-based neutralization test (PVNT) was a kind gift from Renap Therapeutics Co. (Tehran, Iran). Vero 76 cell line (Vero C1008; CRL-1586, Clone E6) was obtained from ATCC. Recombinant spike protein of SARS-CoV-2 with C-terminal histidine tag expressed in Baculovirus insect cells and recombinant RBD of SARS-CoV-2 spike protein with C-terminal histidine tag (RBD-His) expressed in HEK293T cells were purchased from Sino Biological Inc. (Beijing, China). Full-length trimeric spike antigen variants were obtained from BioServUK–CalibreScientific (Sheffield, United Kingdom). Rabbit anti-sheep immunoglobulins (Ig), and horse-radish peroxidase (HRP)-conjugated sheep anti-mouse Ig were purchased from SinaBiotech (Tehran, Iran). Mouse monoclonal antibody isotyping reagents, goat anti-mouse IgG, IgA, and IgM isotype-specific polyclonal antibodies, complete and incomplete Freund’s adjuvants, 2-Mercaptoethanol (2ME), Tween-20, skimmed milk, pristane and all reagents used for cell culture, including Roswell Park Memorial Institute (RPMI) 1640 medium, fetal bovine serum (FBS), L-glutamine, penicillin and streptomycin, HAT supplement (50X), polyethylene glycol (PEG 1500), and dimethyl sulfoxide (DMSO) were purchased from Sigma-Aldrich (Darmstadt, Germany). Anti-nucleocapsid antibody, Alexa Fluor 647-labeled goat anti-rabbit monoclonal antibody, and Sytox green nucleic acid stain were obtained from GeneTex (CA, United States), Abcam (Cambridge, United Kingdom), and Invitrogen (MA, United States), respectively. Streptococcal protein G (SPG) affinity columns and chemiluminescence ECL Prime solution were obtained from GE Healthcare (Nordrhein-Westfalen, Germany). The linear peptides were synthesized by Pepmic company (Jiangsu, China). Tetramethylbenzidine (TMB) and SARS-CoV-2 neutralizing antibody detection kit were obtained from Pishtaz Teb Co. (Tehran, Iran). Maxisorp microtiter ELISA plates and cell culture plates and flasks were purchased from Nunc (Roskilde, Denmark).

### Production of anti-severe acute respiratory syndrome coronavirus 2 receptor binding domain-specific monoclonal antibodies

A total of 6–8 weeks old female BALB/c mice were subcutaneously immunized once with 15 μg and then three times with 7.5 μg of SARS-CoV-2 RBD-His protein in combination with complete and incomplete Freund’s adjuvants, respectively, at 2-week intervals. Blood samples were then taken from the tail vein before each injection and sera were prepared by centrifugation. Hyper-immunization was confirmed by enzyme-linked immunosorbent assay (ELISA). Three to 5 days after intravenous (i.v.) injection of antigen, the spleen was harvested, and hybridomas were generated by fusing the extracted splenocytes with Sp2/0-Ag14 myeloma cell line at a 6:1 ratio using PEG 1500. Only hybridomas were grown in the presence of a selective medium containing hypoxanthine, aminopterin, and thymidine (HAT 1X). Hybridomas were screened and sub-cloned by RBD-specific indirect ELISA and limiting dilution method, respectively to obtain anti-RBD, anti-S final clones. MAb producing final clones (5 × 10^6^ cells/mouse) were intraperitoneally injected into pristane-pretreated BALB/c mice. Ascitic fluids were collected, and the SARS-CoV-2 RBD-specific MAbs were purified using a SPG affinity column.

### Linear peptide synthesis

Amino acid sequences of SARS-CoV-2 RBD were obtained from GenBank under accession number YP_009724390.1 to design the linear peptides. A panel of linear peptides spanning the entire sequence of SARS-CoV-2 RBD (each linear peptide contained 20 amino acid residues with 5 residues overlapping with the adjacent peptides) with purity of more than 90% were used for Pepscan. Lyophilized peptides were dissolved in deionized water and/or DMSO to obtain a stock solution, according to the manufacturer’s instructions. Amino acid sequences of peptide sets are shown in Table 1.

**TABLE 1:** Amino acid sequences of RBD peptide sets used in Pepscan.

No.	Code	Position in spike	Amino acid sequence
1	**P1–20**	319–338	RVQPTESIVRFPNITNLCPF
2	**P16–35**	334–353	NLCPFGEVFNATRFASVYAW
3	**P31–50**	349–368	SVYAWNRKRISNCVADYSV
4	**P46–65**	364–383	DYSVLYNSASFSTFKCYGVS
5	**P61–80**	379–398	CYGVSPTKLNDLCFTNVYAD
6	**P76–95**	394–413	NVYADSFVIRGDEVRQIAPG
7	**P91–110**	409–428	QIAPGQTGKIADYNYKLPDD
8	**P106–125**	424–443	KLPDDFTGCVIAWN 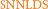
9	**P121–140**	439–458	NNLDSKVGGNYNYLYRLFRK
10	**P136–155**	454–473	RLFRKSNLKPFERDISTEIY
11	**P151–170**	469–488	STEIYQAGSTPCNGVEGFNC
12	**P166–185**	484–503	EGFNCYFPLQSYGFQPTNGV
13	**P181–200**	499–518	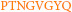 PYRVVVLSFELL
14	**P196–215**	514–533	SFELLHAPATVCGPKKSTNL
15	**P204–223**	522–541	ATVCGPKKSTNLVKNKCVNF

Orange characters show RBM amino acids. RBD, receptor binding domain; RBM, receptor binding motif.

### Enzyme-linked immunosorbent assay

We conducted seven different types of ELISA to evaluate the levels of anti-RBD Ig; in immunized mouse sera, in hybridoma clone supernatants, and to characterize the anti-RBD MAbs:

#### Evaluation of the levels of anti-receptor binding domain and anti-spike antibodies in receptor binding domain-immunized mouse sera and primary hybridoma supernatants

Severe acute respiratory syndrome coronavirus 2 RBD or S antigens, at a concentration of 1 μg/ml, were coated in flat-bottom 96-well microtiter plates in PBS (pH 7.4) overnight at 4°C. After washing with PBST (0.05% v/v Tween-20 in PBS) three times, blocking buffer containing 3% w/v skimmed milk in PBST was added to each well for 1 h at 37°C. Subsequently, mouse sera at 1:1,000, 1:5,000, and 1:25,000 dilutions, or hybridoma supernatants at 1:5 dilution were applied onto the plates in the blocking buffer and incubated at 37°C for 1 h, followed by three washes with PBST. HRP-conjugated sheep anti-mouse Ig antibody at 1:2,000 dilution was used to detect the anti-RBD and anti-S levels in RBD-immunized mouse sera and primary hybridoma supernatants. The reaction was developed by adding TMB substrate solution for 15 min and stopped by addition of 1 M H_2_SO_4_. Then, the optical densities (ODs) of the reactions were measured at 450 and 630 nm using a microplate reader (BioTek, United States).

#### Peptide-based enzyme-linked immunosorbent assay for receptor binding domain-immunized mouse sera, primary hybridoma supernatants, and monoclonal antibodies

Preliminary epitope screening was performed using five pooled RBD peptide sets each composed of three peptides. ELISA was conducted according to the protocol described in section “Evaluation of the levels of anti-receptor binding domain and anti-spike antibodies in receptor binding domain-immunized mouse sera and primary hybridoma supernatants” with the following modifications. In brief, 3.5 μg/ml of each RBD peptide in three peptide pool sets, or 1 μg/ml of RBD were coated onto the ELISA plates in PBS and incubated overnight at 4°C. After washing and blocking serum samples at 1:200 dilution or hybridoma supernatants at 1:5 dilution or MAbs at a concentration of 1 μg/ml in the blocking buffer were added and incubated for another 1 h at 37°C. HRP-conjugated sheep anti-mouse Ig antibody was used for detecting peptide-bound antibodies. Color development was performed as described in section “Evaluation of the levels of anti-receptor binding domain and anti-spike antibodies in receptor binding domain-immunized mouse sera and primary hybridoma supernatants.”

Further assessment procedures were performed to evaluate the reactivity against individual immunodominant linear peptides by immobilizing each peptide into ELISA plates with a concentration of 5 μg/ml. ELISA was conducted according to the protocol described above.

#### Surrogate viral neutralization test

Severe acute respiratory syndrome coronavirus 2 neutralizing antibody detection kit was used for the surrogate viral neutralization test (SVNT) assay according to the manufacturer’s instructions. Briefly, ACE2-HRP was mixed with different concentrations of MAbs, serially diluted mouse sera, or peptide-adsorbed mouse sera and added to RBD pre-coated plates. After incubation for 30 min at 37°C, unbound HRP-conjugated antigens were removed by five PBST washes. After adding TMB and stop solution, the OD was measured by a plate reader.

The percentage of inhibition was determined as follows:

Inhibition(%)=((negativecontrolOD-sampleOD)/negativecontrolOD)×100.

For peptide adsorption, P76–95, P91–110, and P136–155 were selected as the immunodominant peptides. P2-NP (a linear peptide belonging to SARS-CoV-2 nucleocapsid) and P106–125 were also selected as irrelevant peptide and non-reactive control, respectively. First, the immunized mouse sera were serially diluted at a starting dilution of 1:10 and the ID50 values (inhibition dilution of 50%) were calculated in SVNT. Subsequently, 10 μg/ml of selected peptides were incubated with diluted mouse sera at their ID50 dilution point for 2 h at 37°C to adsorb peptide-specific antibodies. Peptides were separately diluted in the sample diluent at a concentration of 10 μg/ml in the absence of mouse serum as peptide controls to assess whether a similar concentration of peptides could individually inhibit the binding of ACE2-HRP to RBD. Peptide-adsorbed mouse sera, non-adsorbed mouse sera, and peptide controls were then mixed with ACE2-HRP and incubated for 30 min at 37°C in RBD pre-coated plates. ELISA was conducted according to the protocol described above.

#### Isotype determination

Severe acute respiratory syndrome coronavirus 2 RBD-specific MAbs (1 μg/ml) were coated into 96-well ELISA plates for 1.5 h at 37°C. After washing with PBST and blocking the remaining binding sites, the plates were incubated with goat anti-mouse IgG, IgA, and IgM isotype-specific polyclonal antibodies (1:1,000) for 20 min at 37°C, followed by washing and incubation with HRP-labeled rabbit anti-sheep Ig (1:3,000) for 20 min at 37°C. After washing the plates, adding TMB solution to the wells, and stopping the enzymatic reaction with 1 M H_2_SO_4_, ODs were measured as described in section “Evaluation of the levels of anti-receptor binding domain and anti-spike antibodies in receptor binding domain-immunized mouse sera and primary hybridoma supernatants.”

#### Monoclonal antibodies affinity determination

The binding affinities of SARS-CoV-2 RBD-specific MAbs were determined using an ELISA-based method (27, 28). Briefly, ELISA plates were coated with recombinant SARS-CoV-2 RBD protein (1,000, 500, 250, 125, 62, and 31 ng/ml) for 1.5 h at 37°C. After washing and blocking, serially diluted MAbs were added and incubated for 1 h at 37°C, followed by washing and incubation with HRP-labeled sheep anti-mouse Ig for 1 h at 37°C. After washing, TMB solution was added to the wells, enzymatic reaction was stopped, and ODs were measured as described in section “Evaluation of the levels of anti-receptor binding domain and anti-spike antibodies in receptor binding domain-immunized mouse sera and primary hybridoma supernatants,” followed by calculating affinity constants (KD) following the given equations (27, 28). Briefly, ODs were plotted against logarithmic values of antibody concentration. Antibody concentration resulting in half of the maximum OD ([Ab]t) at each antigen concentration was chosen for the affinity measurement using the equation KD = (n−1/2) (n[Ab′]t−[Ab]t). n equals [Ag]/[Ag′], where [Ag] and [Ag′] correspond to the higher and lower concentration of antigen. [Ab′]t and [Ab]t correspond to the antibody concentrations giving 50% of maximum OD at [Ag′] and [Ag], respectively. The mean of the calculations for three non-overlapping antigen concentrations was considered as the final KD value.

#### Monoclonal antibodies cross-competition assay

To relatively map the epitope location of SARS-CoV-2 RBD-specific MAbs, the ability of unlabeled MAbs (competitors) to compete with HRP-labeled ones was assessed by a competitive ELISA. 96-well ELISA plates were coated with recombinant SARS-CoV-2 RBD protein (1 μg/ml) for 1.5 h at 37°C. After washing the plates with PBST and treating the wells with blocking buffer, 25 μl of each competitor MAb was mixed with 25 μl of each HRP-labeled MAb to reach the final concentration of 5, 20, and 60 μg/ml of competitor and 1 μg/ml of HRP-labeled MAb. The mixtures were added to RBD-coated plates for 1 h at 37°C. After washing, the enzymatic reaction was developed by TMB solution and stopped by 1 M H_2_SO_4_, followed by measuring ODs. The percentage of competition was determined as follows:

Competition(%)=((HRP-labeledMAbODalone-competitorplusHRP-labeledMAbOD)/HRP-labeledMAbODalone)×100.

#### Reactivity of monoclonal antibodies to severe acute respiratory syndrome coronavirus 2 spike variants

Full-length trimeric spike antigen variants were coated with a concentration of 2 and 0.5μg/ml. After blocking, 1 or 0.25 μg/ml of selected MAbs were added to the plates followed by washing and incubating with HRP-labeled sheep anti-mouse Ig for 1 h at 37°C. The reaction development and stopping were done by TMB and 1 M H_2_SO_4_, respectively.

### Western blotting

Reactivity of selected MAbs against non-reduced or 1% 2ME-reduced form of RBD was assessed by Western blotting. A total of 1 μg of RBD was applied to 10% polyacrylamide gel in SDS sample buffer. Proteins were separated by electrophoresis at 100 V for 1 h and transferred to a 0.45 μm hydrophilic polyvinylidene fluoride (PVDF) membrane at 110 V for 1.5 h. After blocking the membranes in blocking buffer (5% skimmed milk in PBS) overnight at 4°C, the membranes were incubated with 1 μg/ml of MAbs in the blocking solution for 45 min at room temperature (RT). Subsequently, the membranes were washed five times with PBST, then incubated with secondary HRP-conjugated sheep anti-mouse Ig at RT for 45 min, followed by washing five times for 5 min. Finally, positive signals were detected by chemiluminescence ECL Prime solution.

### Pseudovirus-based neutralization test

Angiotensin-converting enzyme-2-expressing HEK293T cells were cultured in RPMI-1640 medium supplemented with 10% FBS, 2 mM L-glutamine, 100 U/ml penicillin, 100 μg/ml streptomycin, and incubated at 37°C, 5% CO_2_ and, 95% humidity. MAbs were diluted in RPMI-1640 medium supplemented with 10% FBS and mixed with the same volume of eGFP-SARS-CoV-2 spike pseudotyped lentivirus corresponding to wild-type (WT, D614G genotype) to reach the final concentration of 15, 5, 0.5, 0.1, and 0.01 μg/ml from each intact MAb in a 96-well plate, followed by incubation at 37°C for 2 h. Subsequently, ACE2-expressing HEK293T cells were detached by trypsin-EDTA 0.25% and added to each well (14 × 10^3^ cells/well). Following incubation for 48 h, the medium was removed. Fluorescence microscopy was used for imaging and detecting the pseudovirus-infected eGFP-positive cells. Microscopic images were taken from at least four microscopic fields. ImageJ software was used to analyze the microscopic images and calculate the fluorescence positive cells. The inhibitory concentration of 50% (IC50) was defined as the Ab concentration leading to a 50% reduction in the percentage of infected cells. IC50 values were determined as described by Ferrara et al. (29).

### Conventional authentic virus-neutralizing assay

Vero 76 cells were plated to a 96-well plate at a density of 12.5 × 10^3^ cells/well, 1 day before the infection. A total of 50 μl of fourfold serially diluted MAbs starting at 20 μg/ml were incubated with 50 μl of WT SARS-CoV-2 strain (D614G genotype), alpha, or omicron strain at MOI of 10, leading to about 30% infected cells and further added to priorly seeded Vero 76 cells. After 48 h, the cells were fixed in methanol for 20 min, washed in PBS, and stained with rabbit anti-nucleocapsid antibody at 1:200 dilution in the perm-wash buffer for 45 min at RT. Subsequently, Alexa Fluor 647-labeled goat anti-rabbit MAb at 1:200 dilution in PBS containing 5% FBS was added and incubated for 45 min at RT. Total cells were detected by Sytox green staining. Total cells (Sytox green positive cells) and infected cells (nucleocapsid positive cells) were counted using SpectraMax MiniMax Imaging Cytometer (Molecular Devices LLC). The percentage of infected cells was recorded and IC50 was calculated as described in section “Conventional authentic virus-neutralizing assay.”

### Statistical analyses

All data were statistically analyzed by Prism v9 (San Diego, CA, United States) and represented as the mean ± SD. Binding inhibition of ACE2 in SVNT by immunodominant peptides or non-reactive and irrelevant peptides-adsorbed RBD-immunized mouse sera was analyzed by ordinary one-way ANOVA. The *p*-values of less than *0.05, **0.01, ***0.001, and ****0.0001) were considered statistically significant. The quantitative cut-off value for positive reactivity of hybridoma supernatants in Pepscan analysis was defined as the mean OD of negative samples plus 3 SDs. Inhibition rates of MAbs and sera were calculated based on the decrease in the fluorescence positive cells (for pseudotype-based neutralization assay) or the decrease in nucleocapsid positive cells (for live SARS-CoV-2-based neutralization assay). IC50 value (inhibition concentration 50%) for MAbs or ID50 value (inhibition dilution 50%) for sera were calculated using non-linear regression, i.e., (inhibitor) vs. response (four parameters). Correlation between SVNT, PVNT, and conventional virus neutralization test (CVNT) was analyzed by Spearman test. Fold change was computed as the ratio of the changes between final IC50 values of MAbs against variants of concern (VOCs) (Y) and the original IC50 values of MAbs against WT, D614G genotype (X) over the initial value using the following equation: *Foldchange* = (Y−X)/X.

## Results

### Titration of anti-receptor binding domain and anti-spike antibodies in receptor binding domain-immunized mouse sera

**FIGURE 1:**
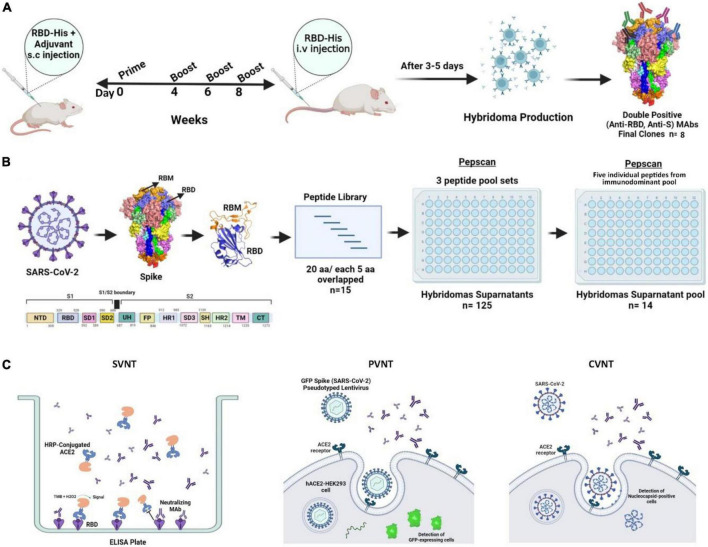
Schematic representation of mouse immunization and hybridoma production workflow to generate and characterize anti-SARS-CoV-2-RBD MAbs. **(A)** A total of 6–8-week old female BALB/c mice were immunized with RBD-His and complete Freund’s adjuvant on day 0 followed by boosts with RBD-His and incomplete Freund’s adjuvant at weeks 4, 6, and 8. Blood samples were collected for ELISA before each injection. A total of 3–5 days after intravenous injection of RBD, mice were sacrificed and splenocytes were fused with Sp2/0 cells. Finally, eight clones were generated with reactivity against both RBD and S antigens. **(B)** SARS-CoV-2 RBD Pepscan was performed using a panel of linear peptides spanning the entire sequence of SARS-CoV-2 RBD of spike containing 20 amino acid residues with 5 residues overlapping with the adjacent peptides for the characterization of hybridoma supernatants and purified MAbs. First, the reactivity of hybridoma supernatants was assessed against three peptide pool sets. Subsequently, the linear peptide-reacting hybridoma supernatants were pooled and their reactivity was assessed against individual peptides of the immunodominant peptide pool. **(C)** SVNT, PVNT, and CVNT were performed to characterize MAbs. aa, amino acid; ACE2, angiotensin-converting enzyme-2; CVNT, conventional virus-neutralizing test; ELISA, enzyme-linked immunosorbent assay; HRP, horse-radish peroxidase; i.v., intravenous; MAb, monoclonal antibody; PVNT, pseudovirus neutralizing test; RBD, receptor binding domain; RBM, receptor binding motif; S, spike; SARS-CoV-2, severe acute respiratory syndrome coronavirus 2; s.c., subcutaneous; SVNT, surrogate virus-neutralizing test.

**FIGURE 2:**
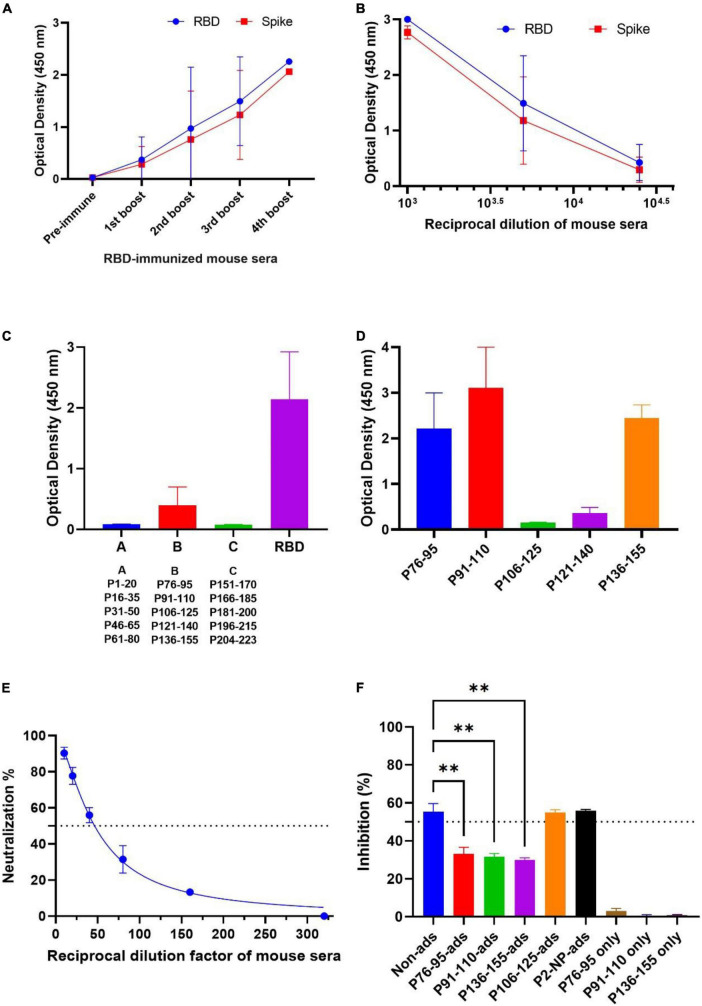
Antibody responses in the immunized mouse sera against spike, RBD, and linear peptides by ELISA and peptide-adsorption assay by SVNT. **(A)** Antibody responses against RBD and spike in the immunized mouse sera during the immunization schedule. Sera were tested by ELISA at 1:5,000 dilution. **(B)** Titration of sera collected from the sacrificed mice before the spleen harvest against RBD and S proteins. **(C)** The immunodominant epitopes of the SARS-CoV-2 RBD protein were mapped by Pepscan analysis against mouse immunized sera and the immunodominant peptide pool was identified. Mouse hyperimmune sera (1:200) were subjected to three peptide pools (A–C) of overlapping peptides and RBD antigen by ELISA. Each pool contained five 20-mer peptides spanning the entire RBD domain of the spike. **(D)** The immunodominant epitopes among “pool B” peptides from the RBD protein was identified by ELISA. Individual peptides (P76–95, P91–110, P106–125, P121–140, and P136–155) were coated to screen the serum samples from hyperimmune mice to determine the most reactive peptides. **(E)** Titration curves of immunized mouse sera were presented in SVNT by testing serially diluted sera at a starting dilution of 1:10. The dotted lines on each graph indicate 50% inhibition. **(F)** Binding inhibition of ACE2 in SVNT by immunodominant peptides (P76–95, P91–110, and P136–155) or non-reactive and irrelevant peptides (P106–125 and P2–NP)-adsorbed RBD-immunized mouse sera. Peptides were separately used in the absence of mouse sera as peptide controls. Data analyzed by ordinary one-way ANOVA **p < 0.01. The solid lines represent mean values ± SD. ACE2, angiotensin-converting enzyme-2; ELISA, enzyme-linked immunosorbent assay; NP, nucleocapsid; RBD, receptor binding domain; S, spike; SARS-CoV-2, severe acute respiratory syndrome coronavirus 2; SVNT, surrogate virus-neutralizing test; ads, adsorbed sera.

We employed RBD protein with C-terminal histidine tag (RBD-His) as an immunogen to immunize BALB/c mice (the study design is depicted in Figure 1). The mice were primed with RBD-His in combination with complete Freund’s adjuvant on day 0 and boosted with RBD-His and incomplete Freund’s adjuvant at weeks 4, 6, and 8. Bleeding was done before each injection and 4 weeks after the last booster dose (Figure 1). Titration of hyperimmune mouse sera against RBD and S proteins by ELISA showed a mean half-maximal effective serum titer of 6,000 and 7,000 against spike and RBD, respectively, 3–5 days after i.v. booster administration of RBD (Figures 2A,B).

### Pepscan analysis of receptor binding domain-immunized mouse serum

To map the linear epitopes recognized by mouse polyclonal anti-RBD antibodies, a series of 15 overlapping peptides covering the RBD sequence of the S protein were used as coating antigens in ELISA (Figures 1B, 2C,D). First, three pooled peptide sets (peptide pools A, B, and C) were employed for Pepscan. Preliminary Pepscan analysis indicated one dominant peptide pool (pool B), including amino acids 76–155 (Figure 2C). Further Pepscan assay performed on individual linear peptides showed relatively high reactivity to peptides P76–95, P91–110, and P136–155 in comparison with P106–125 and P121–140 (Figure 2D). P136–155 is located in RBM, which is critical for ACE2 binding (8), while P76–95 and P91–110 are located in the core subdomain in the N-terminal of RBD.

### Adsorption of immunized mouse sera with immunodominant linear peptides

To evaluate the neutralizing effect of antibodies against the immunodominant linear peptides identified by Pepscan, serum adsorption assays with P76–95, P91–110, and P136–155 were performed by SVNT. The immunized mouse sera showed ID50 of about 1:50 dilution in SVNT (Figure 2E). We found that sera adsorbed with P76–95, P91–110, or P136–155 peptide could significantly reduce binding ability of ACE2 to RBD compared with the non-adsorbed serum controls (*p* < 0.01) (Figure 2F), implying that the antibodies targeting these three immunodominant linear epitopes contribute to the anti-RBD neutralizing response.

### Isolation and characterization of receptor binding domain-specific monoclonal antibodies

Preliminary growing hybridomas were screened against RBD by ELISA. A total of 125 RBD-reactive hybridomas were initially identified and assessed by Pepscan. Subsequently, eight stable hybridomas producing double reactive (anti-RBD and anti-S) MAbs were selected, cloned, and characterized.

### Epitope mapping of anti-receptor binding domain hybridoma supernatants

**FIGURE 3:**
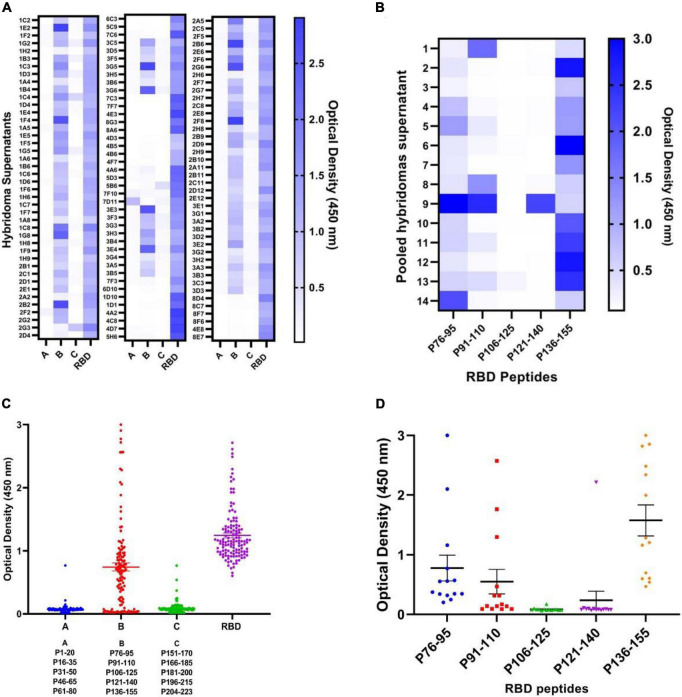
Pepscan analysis of hybridoma supernatants by ELISA. **(A,B)** Mapping the immunodominant epitopes of the SARS-CoV-2 RBD protein by Pepscan analysis against hybridoma supernatants and identification of the immunodominant peptide pools. A total of 125 anti-RBD hybridoma supernatants were subjected to three pools **(A–C)** of overlapping peptides and RBD antigen by ELISA. Each pool contains five 20-mer overlapping peptides covering the entire RBD. Reactivities are presented as heatmap and dot plot. **(C,D)** Identification of the immunodominant epitope among “pool B” peptides from the RBD protein. Individual peptides (P76–95, P91–110, P106–125, P121–140, and P136–155) were screened with hybridoma supernatants to determine the most reactive peptide. Reactivities are presented in the heatmap and dot plot. The solid lines represent mean ± SD. ELISA, enzyme-linked immunosorbent assay; RBD, receptor binding domain; S, spike; SARS-CoV-2, severe acute respiratory syndrome coronavirus 2.

To determine epitope specificity of the 125 initial RBD-reactive hybridomas as well as the final eight selected clones, three peptide pool sets designated A, B, and C, each composed of five consecutive peptides, were employed and tested by ELISA (Figure 3). The results showed that most of the hybridoma supernatants (*n* = 70) react with peptide pool B encompassing amino acids 76–155 of the RBD sequence (Figures 3A,B). These findings are consistent with the results obtained from the RBD-immunized mouse sera. Subsequently, peptide pool B-reactive hybridoma supernatants were mixed in 14 pool sets each consisting of five supernatants to evaluate their reactivities against each individual peptide of pool B. Our data demonstrated that a large number of the hybridoma pools sets were reactive to P76–95 (12 out of 14 pool sets) and P136–155 (14 out of 14 pool sets) peptides, which are located in the core sub-domain and RBM of RBD, respectively (Figures 3C,D). Due to shortage of materials, we could not test all the positive hybridoma supernatants individually against the five peptides of the pool B. Finally, eight hybridomas reactive with both RBD and S proteins, designated 1D1, 1D10, 2C5, 2D9, 2F8, 2G3, 3B6, and 3G5 were stable and could be cloned and characterized. Based on Pepscan results, 2F8 MAb reacts with the peptide P136–155 (RLFRKSNLKPFERDISTEIY), which is located in RBM of RBD (8) and 3G5 MAb reacts with the peptide P76–95 (NVYADSFVIRGDEVRQIAPG), which is located in the N-terminal region of the RBD (Table 1). However, despite ELISA binding to the spike and RBD, the remaining 6 MAbs failed to react with any of the peptides.

### Affinity and isotype

**FIGURE 4:**
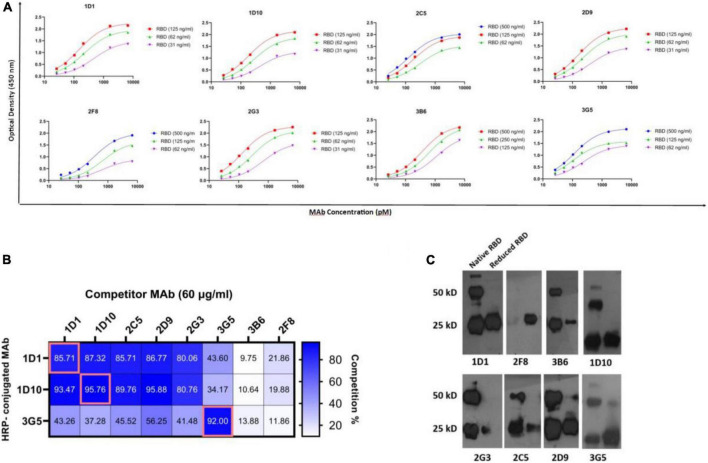
Binding affinity, competition profile, and structural characterization of anti-RBD MAbs. **(A)** The binding affinity of MAbs to RBD protein was measured by an ELISA-based method. MAbs concentration at OD 50% was used to calculate affinity constants (Ka) between each pair of sigmoidal curves in the graphs based on the equations referred to in section “Materials and methods.” The final Ka was obtained by averaging three calculated Ka. **(B)** The competition profile of MAbs was determined by competitive ELISA. Results of competition ELISA are presented as the percentage of competition by the competitor MAbs compared with no-competitor groups. Competition for more than 50% was considered as positive. **(C)** Western blot representative of reactivity of selected double-reactive MAbs against 2ME-reduced and native RBD proteins. A total of 25 and 50 kD bands show monomeric and dimeric RBD protein. Ag, antigen; ELISA, enzyme-linked immunosorbent assay; HRP, horse-radish peroxidase; MAb, monoclonal antibody; nM, nanomolar; OD, optical density; pM, picomolar; RBD, receptor binding domain.

Representative binding affinity curves obtained for RBD-specific MAbs are presented in Figure 4A. The affinity constants were found to be in the range of 0.36–1.71 nM, the highest and lowest affinities belonged to 3G5 and 2F8 MAbs, respectively. The isotype was found to be IgG for all the MAbs (Table 2).

**TABLE 2:** Characteristics of anti-RBD MAbs.

No.	MAbs designation	Isotype	Affinity (nM)	Peptide reactivity	SVNT (inhibition %) 40 μ g/ml	PVNT (neutralization %) 15 μ g/ml	CVNT (neutralization %) 10 μ g/ml	SVNT (IC50 μ g/ml)	PVNT (IC50 μ g/ml)	CVNT* (IC50 μ g/ml)	IC50 fold change against omicron
1	**1D1**	IgG	0.49	–	100	100	100	8.5	1.8	0.1	64
2	**1D10**	IgG	0.57	–	100	100	100	9.2	0.9	0.1	62
3	**2C5**	IgG	0.51	–	87	56	100	14.5	12.4	0.3	12
4	**2D9**	IgG	0.53	–	100	100	100	6.3	1.6	0.2	22
5	**2F8**	IgG	1.71	P136–155	0	0	0	–	–	–	–
6	**2G3**	IgG	0.76	–	0	0	8	–	–	–	–
7	**3B6**	IgG	1.06	–	44	32	100	43.1	32	0.2	23.5
8	**3G5**	IgG	0.36	P76–95	8	28	100	48	38	0.6	18.7

*IC50 values were expressed as IC50 mean of three independent experiments. CNVT, conventional virus neutralization test; IgG, immunoglobulin G; MAb, monoclonal antibody; nM, nanomolar; PVNT, pseudovirus virus neutralization test; RBD, receptor binding domain; SVNT, surrogate virus neutralization test. The bold terms in the second column are the MAbs designations.

### Cross-competition of monoclonal antibodies for binding to receptor binding domain

Three MAbs (1D1, 1D10, and 3G5) were randomly HRP-conjugated and their binding activities to RBD was measured in the presence or absence of the other unlabeled anti-RBD MAbs, as competitors. Accordingly, three distinct groups of MAbs were identified. Five MAbs, including 1D1, 1D10, 2C5, 2D9, and 2G3 were classified into one group, since they competed with two of the HRP-conjugated MAbs (1D1 and 1D10) with a similar pattern. The binding of 3G5 MAb to RBD, which recognizes a distinct linear epitope (P76–95), is partially inhibited by five other MAbs, including 1D1, 1D10, 2C5, 2D9, and 2G3, implying that 3G5 binds to an epitope in the vicinity of the epitope(s) recognized by these five MAbs. The other two MAbs (3B6 and 2F8) displayed different cross-competition profile (Figure 4B). These results demonstrate that our MAbs recognize multiple epitopes and are not limited to a few immunodominant epitopes in RBD.

### Western blotting analysis of the monoclonal antibodies

The reactivities of the MAbs with non-reduced and 2ME-reduced RBD proteins were assessed by Western blotting (Figure 4C). Due to the overload of protein in the gel and the high intensity of the bands, we considered the pale bands as non-specific (pale bands in 50 kD of reduced RBD, corresponding to 2D9 and 3G5 MAbs, as well as pale bands in 25 kD of reduced RBD, corresponding to 3B6, 2G3, and 2C5 MAbs). Although 1D1, 1D10, and 2D9 were not reactive to any of the linear peptides in Pepscan, they were reactive against both non-reduced and 2ME-reduced RBD proteins, suggesting that their epitope is a disulfide bond-independent conformational epitope. It is possible that 1D1, 1D10, and 2D9 (but not 2C5 and 2G3) might be originated from a common clone based on their reactivity obtained from competition ELISA and Western blotting. However, 2G3, 2C5, and 3B6 did not react with 2ME-reduced RBD protein, which indicates that they recognize conformational disulfide bond-dependent epitopes. 3G5, as a linear peptide-reactive MAb (P76–95), showed reactivity against both non-reduced and 2ME-reduced RBD protein. The last MAb (2F8) which reacted with the linear peptide P136–155 in ELISA, recognized the 2ME-reduced, but not non-reduced form of RBD protein which suggests reactivity against a disulfide-bond dependent linear epitope. Two-band pattern of the non-reduced form of recombinant RBD with MW sizes of 25 KD and 50 KD, is observed in almost all developed lanes, which is most likely the result of RBD dimerization (Figure 4C).

### Wild-type severe acute respiratory syndrome coronavirus 2 virus neutralization assessment

Surrogate viral neutralization test, PVNT, and CVNT assays were employed to assess the neutralizing potential of the eight selected RBD-specific MAbs generated in this study. In the first step, a single high concentration of all eight selected MAbs was used for the SVNT (40 μg/ml), PVNT (15 μg/ml), and CVNT (10 μg/ml) assays to assess the neutralization potency of the MAbs. As shown in Table 2 and based on the preliminary SVNT, PVNT, and CVNT results, four MAbs, including 1D1, 1D10, 2C5, and 2D9 belonging to a cross-reactive group, demonstrated neutralization capacity in all assays. Two MAbs, 3B6 and 3G5, showed weak neutralization activity (based on SVNT and PVNT results), while the remaining two MAbs (2F8 and 2G3) did not show any neutralization activity at the highest concentrations mentioned above. The six neutralizing MAbs, including 1D1, 1D10, 2C5, 2D9, 3B6, and 3G5, were further tested to determine their IC50 in SVNT, PVNT, and CVNT.

**FIGURE 5:**
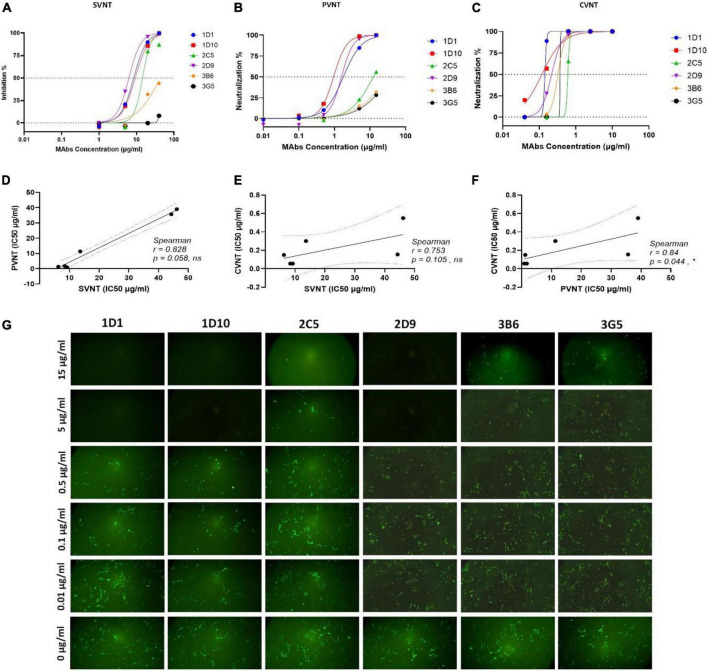
Neutralizing activity of MAbs was determined by PVNT, SVNT, and CVNT. **(A)** Selected RBD-specific MAbs were assessed for neutralization by SVNT. HRP-conjugated ACE2 protein was used to determine its binding to immobilized RBD in the presence of MAbs at the concentrations of 1, 5, 20, and 40 μg/ml. The percentage (%) of inhibition was calculated based on the OD of HRP-conjugated ACE2 with or without presence of RBD-specific MAbs. Dose-response curves are presented for each MAb. The IC50 value was calculated by non-linear regression (four-parameter), as represented in Table 2. **(B)** Dose-response curves of selected MAbs determined by PVNT. Five different concentrations of selected MAbs, 0.01, 0.1, 0.5, 5, and 15 μg/ml, were incubated with pseudovirus and added to HEK293T-hACE2 cells (14 × 10^3^ cells/well). ImageJ software was used to calculate the fluorescence positive cells 60 h post-infection to calculate IC50 values and the inhibition ratios. The IC50 was calculated by non-linear regression (four-parameter), as represented in Table 2. **(C)** Representative dose-response curves of selected MAbs determined by CVNT. The selected MAbs were diluted to different concentrations, incubated with the WT D614G SARS-CoV-2 to reach the final concentrations of 0.04, 0.16, 0.63, 2.5, and 10 μg/ml, and added to Vero 76 cells. The percentage of infected cells was calculated by counting nucleocapsid positive cells versus total cells. The IC50 was calculated by non-linear regression (four-parameter), as represented in Table 2. The dotted lines on each graph indicate 0 and 50% neutralization. **(D–F)** The correlation of obtained IC50 values between SVNT, PVNT, and CVNT was calculated by Spearman analysis. **(G)** Representative fluorescence images of HEK293T cells expressing hACE2 after infection with eGFP-pseudotyped lentiviruses in the absence or presence of different concentrations of anti-RBD MAbs. ACE2, angiotensin-converting enzyme-2; CVNT, conventional virus-neutralizing test; HRP, horse-radish peroxidase; IC50, inhibition concentration 50%; MAb, monoclonal antibody; OD, optical density; PVNT, pseudovirus neutralization test; RBD, receptor binding domain; SARS-CoV-2, severe acute respiratory syndrome coronavirus 2; SVNT, surrogate virus-neutralizing test.

Using the SVNT assay, all these MAbs were found to dose-dependently block ACE2 binding to RBD, with IC50 values ranging from 6.3 to 48 μg/ml in SVNT (Figure 5A and Table 2). While the PVNT assay using SARS-CoV-2 pseudovirus (wild-type, D614G genotype) and human ACE2-overexpressing HEK293T cells gave IC50 values ranging from 0.9 to 38 μg/ml (Figures 5B,G and Table 2), the authentic CVNT assay using wild-type SARS-CoV-2 (wild-type, D614G genotype) and Vero 76 cell lines demonstrated IC50 values ranging from 0.1 to 0.6 μg/ml (Figure 5C and Table 2). Representative PVNT results are illustrated in Figure 5G. The IC50 results obtained from all three neutralization assays are depicted in Table 2. Among the six neutralizing MAbs, 1D1 and 1D10 were the most potent and 3G5 was the weakest in terms of neutralization potency (based on PVNT and CVNT results). Positive correlation for the IC50 values was observed between all three neutralization assays. While PVNT and CVNT demonstrated the highest correlation (*p* = 0.044), SVNT and CVNT (*p* = 0.105), and SVNT and PVNT (*p* = 0.058) displayed lower correlation (Figures 5D–F).

### Neutralizing capacity of monoclonal antibodies on alpha and omicron variants

**FIGURE 6:**
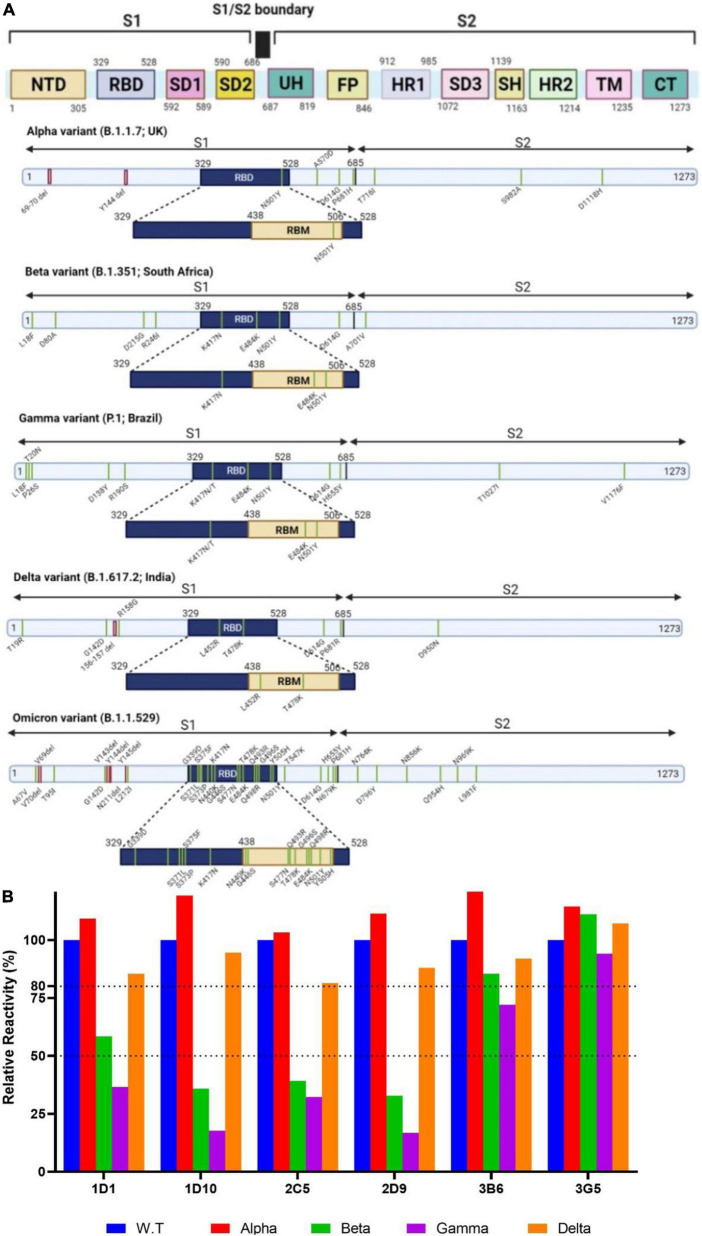
Schematic overview of the spike proteins from the SARS-CoV-2 VOCs and binding activity of MAbs against VOCs. **(A)** The primary structure of the spike protein and the location of the mutations in the context of the spike protein are shown in distinct panels. **(B)** Relative binding reactivity of selected MAbs was measured against the trimeric spike of SARS-CoV-2 alpha (red), beta (green), gamma (purple), and delta (brown) variants compared with that of wild-type (blue) by ELISA. ELISA, enzyme-linked immunosorbent assay; FP, fusion peptide; HR, heptad repeat; MAb, monoclonal antibody; NTD, N-terminal domain; RBD, receptor binding domain; RBM, receptor-binding motif; S, spike; SARS-CoV-2, severe acute respiratory syndrome coronavirus 2; SD, subdomain; SH, stem helix; TM, transmembrane; UH, upstream helix; VOC, variant of concern.

Severe acute respiratory syndrome coronavirus 2 VOCs are differentially mutated in different regions of the spike protein (Figure 6A). We initially evaluated binding reactivity of the selected neutralizing MAbs to the trimeric spike of SARS-CoV-2 VOCs (alpha, beta, gamma, and delta) compared with that of wild-type virus by ELISA. Our results showed no substantial increased or decreased reactivity of MAbs against alpha variant. Relative reactivities of 1D1, 1D10, 2C5, and 2D9 declined by more than 50% against beta and gamma variants. However, 3B6 and 3G5 revealed no substantial decline against beta and gamma variants (Figure 6B). We did not have access to the recombinant spike protein of omicron variant to perform the experiment and compare the results.

**FIGURE 7:**
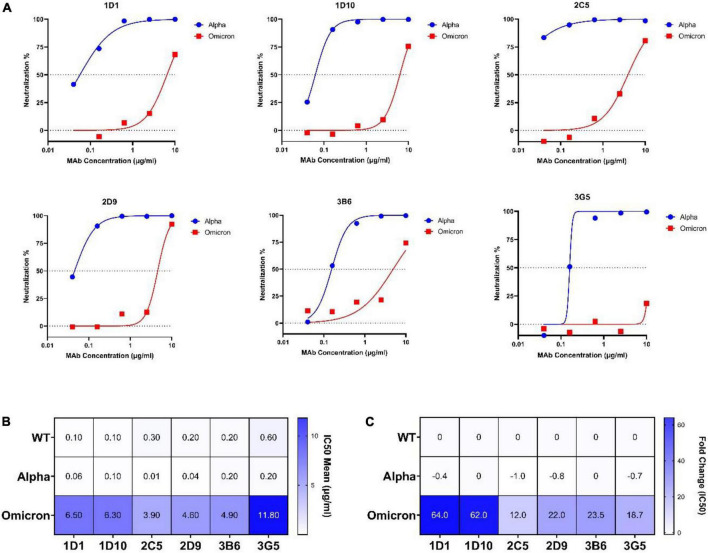
Neutralization potency of selected MAbs against alpha and omicron variants. **(A)** Dose-response curves and IC50 values of selected MAbs at different concentrations (0.04, 0.16, 0.63, 2.5, and 10 μg/ml) against alpha and omicron variants determined by CVNT. The dotted lines on each graph indicate 0 and 50% neutralization. The IC50 was calculated by non-linear regression (four-parameter), as represented in **(B)**. **(B)** Heatmap of IC50 mean values of selected MAbs against WT (wild-type), alpha, and omicron viruses. **(C)** Heatmap of calculated IC50 fold changes of selected MAbs against alpha and omicron variant obtained from CVNT based on IC50 values against WT virus. The increase or decrease in IC50 values relative to WT is shown as positive or negative fold change values, respectively. CVNT, conventional virus-neutralizing test; IC50, inhibition concentration 50%; MAb, monoclonal antibody; VOC, variant of concern.

The neutralization efficiency of our MAbs against the omicron variant was assessed by CVNT assay in parallel with the alpha variant (Figure 7A). Consistent with our ELISA results, minor or no changes were found in IC50 values of MAbs against the alpha variant (Figures 7B,C). However, a substantial reduction of neutralization activity to the omicron variant was found for all MAbs. Specifically, the IC50 values of 1D1 and 1D10 increased 64- and 62-fold (IC50: 6.5 and 6.3 μg/ml, respectively), and the IC50 values of 2D9, 3B6, and 3G5 increased 22, 23, and 18-fold, respectively. Among the selected MAbs, 2C5 showed the smallest drop of neutralizing activity (IC50: 3.9 μg/ml) against omicron, with about 12-fold increase of IC50 compared to the WT virus (Figures 7B,C).

## Discussion

Coronavirus disease 2019 pandemic has caused serious public health crisis during the last 2 years. Development of prophylactic and therapeutic MAbs may help to protect the patients at high risk of progression to severe COVID-19 against more pathogenic or transmissible SARS-CoV-2 variants. The S protein of SARS-CoV-2 is a key protein responsible for binding to ACE2. The RBD is the major immunodominant and immunoprotective region of the S protein which elicits potent virus neutralizing antibodies and has been used for the design and development of SARS-CoV-2 vaccines (30–32). Such neutralizing antibodies are widely detected in serum of COVID-19 patients (33–35). RBM is a part of RBD which directly binds to the ACE2 receptor and could be considered as a promising target for generating neutralizing antibodies (36).

In the present study, we generated and characterized a panel of murine neutralizing MAbs against SARS-CoV-2 using recombinant RBD protein as the immunogen (Figure 1). This approach has also been used in other studies (37–41). We investigated epitope specificity of the serum antibody as well as all preliminary growing hybridomas by Pepscan. Our results showed that the antibody response in BALB/c mice is largely directed against linear epitopes. These findings are different from our previous results obtained from COVID-19 convalescent sera which showed that the antibody response is mainly directed against conformational epitopes of RBD in human (42).

Our Pepscan results showed that P76–91, P91–110, and especially P136–155 peptides account for a fraction of the neutralizing antibody pool in immunized mouse sera based on our peptide adsorption assays (Figures 2C–F). This finding is consistent with a recent study in which epitope profiling was performed on a panel of sera from RBD-immunized mice. They demonstrated immunogenicity of R465 (overlapping with P136–155) and R405 (overlapping with P76–95) peptides (41). In another study, 33 predicted linear epitopes of spike were applied to immunize BALB/c mice of which two peptides (S406–420 and S455–469) overlapping with our P76–110 (aa 394–428 of spike) and P136–155 (aa 454–473 of spike) peptides, elicited robust antibody responses against S protein (43).

Interestingly, the P136–155 (RLFRKSNLKPFERDISTEI) peptide, highly recognized by our serum samples and hybridoma supernatants (Figures 2, 3), is located in RBM according to structural analysis of the RBD-ACE2 complex and was shown to elicits neutralizing antibodies in COVID-19 patients (36). One of our final selected MAbs (2F8) reacted with this peptide. Although 2F8 binds to this peptide, it possesses a low affinity (1.71 nM), which may account for its lack of neutralization activity.

The other peptide-reactive MAb, 3G5, which recognizes P76–95 (NVYADSFVIRGDEVRQIAPG) peptide, located in the core subdomain of RBD (aa 394–413), displayed weak neutralizing potency against WT SARS-CoV-2 pseudovirus. Recent studies have reported two MAbs, CB6 and B38, recognizing residues within P76–95 as well as RBM, which displayed neutralizing activity and were able to completely abolish ACE2/RBD binding (44, 45). These MAbs may probably elicit steric hindrance or allosteric effects for binding to ACE2.

Five of our MAbs, including 1D1, 1D10, 2C5, 2D9, and 2G3, displayed similar binding competition patterns, but different neutralizing activities (Figures 4B, 5). These five MAbs showed different epitope specificity in immunoblotting assay (Figure 4C). While 1D1, 1D10, and 2D9 recognized both reduced and non-reduced RBD, 2C5 and 2G3 did not bind to the reduced form of RBD. We speculate that the orientation of binding of these antibodies to their target epitopes may influence their neutralizing capacity, a finding also reported by other investigators (46, 47).

The emergence of immune resistant variants harboring escape mutations in response to the immune pressure is an important issue that must be taken into consideration to control COVID-19. Emerging variants are categorized as either variants of interest (VOI) or VOC (48). Five variants are classified as VOC, including alpha variant (B.1.1.7) containing N501Y substitution in RBD, beta variant (B.1.351) containing three important mutations in RBD, including N501Y, E484K, and K417N, gamma variant (P.1) with biologically important mutations in the RBD region, including N501Y, E484K, and K417N/T, delta variant (B.1.617.2) harboring two substitutions in RBD, including L452R and T478K associated with its higher transmissibility (49–52), and omicron variant (B.1.1.529) harboring 34 mutations, 15 of which are in the RBD region, leading to fourfold increased infectivity compared with the WT SARS-CoV-2 (Figure 6A). Alpha and beta variants are significantly more transmissible (43–82 and 50%, respectively), compared to Wuhan SARS-CoV-2 virus (53, 54), due to N501Y substitution that enhances the accessibility of RBD and binding affinity to ACE2 (53, 55–57). Although K417N/T substitutions found in beta and gamma variants decreased the binding affinity, N501Y and E484K mutations enhanced the binding affinity of their RBDs to ACE2 (58).

Recently, an extensive study was conducted on MAbs authorized for emergency use by the FDA-EU to assess their neutralizing activities against the current VOCs. The results showed slightly reduced neutralizing activity of sotrovimab against the alpha variant due to N501Y mutation. Accordingly, the neutralizing potency of bamlanivimab and casirivimab was completely or significantly lost against the beta variant because of E484K and K417N substitutions. Bamlanivimab lost the neutralization effect against beta variant carrying the E484K substitution (59). Another study reported that yeast expressing mutant RBD harboring the E484K and K417N/T substitutions escaped bamlanivimab and etesevimab, respectively (60). However, imdevimab maintained its neutralization activity against alpha and beta variants (61). Therefore, the emergence of mutations similar to alpha and beta variants is considered an important challenge for therapeutic MAbs. In accordance with these findings, our neutralizing MAbs displayed similar ELISA binding and neutralization against WT and alpha strains (Figures 6B, 7), indicating that our MAbs are insensitive to the N501Y mutation of alpha variant. Of note, the binding of 1D1, 1D10, 2C5, and 2D9 to the trimeric spike of beta and gamma variants showed a substantial decline compared with the WT trimeric spike (Figure 6B), implying contribution of K417 and E484 mutations in these two variants. Similarly, a panel of anti-RBD human MAbs showed decreased binding reactivity and neutralizing activity to spike and authentic virus of beta and gamma variants, as compared to the alpha variant (62). Analysis of a panel of MAbs, including COV2-2196, COV2-2130, sotrovimab, casirivimab, imdevimab, bamlanivimab, and etesevimab, demonstrated no significant changes in neutralizing activity against beta and delta variants, except for imdevimab and bamlanivimab, which displayed 10-fold decrease and complete loss of binding against the delta variant, respectively (63). Our ELISA results indicate that none of our MAbs lost substantial binding against the delta trimeric spike (Figure 6B), implying that in contrast to mutations in RBD of beta and gamma variants, the substitutions in delta variant, including L452R and T478K, might not be critical for reactivity of our MAbs. A chimeric MAb with low binding and neutralization potency against the spike proteins of alpha, beta, and gamma variants has recently been reported which binds and neutralizes the delta variant potently (64). Based on our knowledge from the literature, there is an association between binding ability of MAbs to the critical residues of spike protein of VOCs and their neutralizing potency (62, 64). However, we cannot attribute this association to our MAbs, unless we confirm it by PVNT or CVNT assays. Unfortunately, we could not check the neutralization potency of our MAbs against beta, gamma, and delta variants, because we had no access to these variants.

Assessment of the neutralization potency of MAbs against the latest VOC Omicron is key due to its extensive mutations within the RBD and S proteins. In our panel of MAbs, 1D1 and 1D10, which showed potent neutralization activity against the authentic WT virus, were still able to neutralize the omicron variant, although a more than 70-fold increase of IC50 was observed. Notably, 2C5 with a higher IC50 value against the WT virus was more resilient to the authentic omicron variant (16-fold increase of IC50) compared to 1D1 and 1D10 (Figure 7). Recent study has reported that sotrovimab revealed a threefold reduction, the combination of COV2-2130 and COV2-2196 showed a ∼200-fold decline, and casirivimab, imdevimab, bamlanivimab, etesevimab, and CT-P59 completely lost the neutralizing function against omicron (10). Interestingly, sotrovimab maintained neutralizing potency against the omicron variant (65).

## Conclusion

We produced and characterized new SARS-CoV-2 RBD-specific murine MAbs exhibiting distinct epitope binding and neutralization potency against different VOCs of SARS-CoV-2. Our MAbs, therefore, represent new additional MAb candidates for SARS-CoV-2 inhibition. We are planning to chimerize a number of our potent neutralizing MAbs, to be able to compare their functional and structural properties with their mouse counterparts and to evaluate their therapeutic effects in a preclinical and clinical settings. Understanding the interactions between these MAbs and the RBD epitopes and identifying engaged residues is required for the design of new immunogens for the development of new generation of vaccines protective for a broad spectrum of SARS-CoV-2 variants. The anti-RBD MAbs could also potentially be used to design highly specific and sensitive immunoassays to detect viral particles of SARS-CoV-2 variants in patients samples as a diagnostic tool, in combination with MAbs against other structural proteins of SARS-CoV-2, such as nucleoprotein. However, due to the extensive mutations accumulated in the spike and RBD proteins progressively, as opposed to the anti-nucleoprotein MAbs, the anti-RBD MAbs lose their affinity and reactivity which limits their application for diagnostic purposes.

## Data availability statement

The original contributions presented in this study are included in the article/supplementary material, further inquiries can be directed to the corresponding authors.

## Ethics statement

The animal study was reviewed and approved by the Research Ethics Committee of Tehran University of Medical Sciences (IR.TUMS.SPH.REC.1400.167) and the National Institute for Medical Research Development (NIMAD) of Iran (IR.NIMAD.REC.1399.194).

## Author contributions

FM performed the assays and wrote the original draft. JK, MM, HA, SS, and GL contributed to the performing the assays. A-HZ, VS, GK, AG, and MJ-T contributed to the conceptualization and design of the study. MJ-T reviewed and edited the manuscript. MA, CM, and FS contributed to the project conceptualization, data validation, project administration, supervision, and reviewing and editing of the manuscript. All authors have read and agreed to the published version of the article.
